# Effect of Ammonium Halide Additives on the Performance of Methyl Amine Based Perovskite Solar Cells

**DOI:** 10.3390/ma11081417

**Published:** 2018-08-13

**Authors:** Do Yeon Heo, Zhengtang Luo, Soo Young Kim

**Affiliations:** 1 School of Chemical Engineering and Materials Science, Chung-Ang University, 84 Heukseok-ro, Dongjak-gu, Seoul 06974, Korea; doyoun0312@naver.com; 2 Department of Chemical and Biomolecular Engineering, the University of Hong Kong Science and Technology, Clear Water Bay, Kowloon, Hong Kong

**Keywords:** perovskite structure, perovskite solar cells, additive, ammonium halide

## Abstract

CH_3_NH_3_PbI_3-x_Cl_x_ species were fabricated as light-absorbing layers for perovskite solar cells (PSCs), by employing NH_4_I, NH_4_Br, and NH_4_Cl as additives via annealing at 100 °C for different times. Solutions containing CH_3_NH_3_I, PbI_2_, and PbCl_2_ (4:1:1 molar ratio) in *N*,*N*-dimethylformamide were used to prepare perovskites with NH_4_I, NH_4_Br, and NH_4_Cl as additives, at concentrations of 0.1 M and 0.3 M. The additives helped increase the grain size and reduce pinholes in the perovskite films, as confirmed by field-emission scanning electron microscopy. The X-ray diffraction profiles of CH_3_NH_3_PbI_3-x_Cl_x_ clearly showed peaks at 14° and 28° for the samples with additives, indicative of crystallinity. The best PSC performance with a power conversion efficiency of 9.13%, was achieved using 0.1 M NH_4_I by annealing for 5 min, whereas the power conversion efficiency of the perovskite solar cells without additives was 5.40%.

## 1. Introduction

In 2009, perovskite was first used as a sensitizer for light absorption in dye-sensitized solar cells (DSSCs), which gave a power conversion efficiency (PCE) of 3.8% [1]. Perovskite has an ABX_3_ structure, where A, B, and X represent an organic cation, metal cation, and halide ion, respectively. Given perovskites have high absorption coefficients in the visible region, perovskite nanocrystals, especially CH_3_NH_3_PbX_3_ (X = Br, I, Cl), are used as light absorbers. Park et al. achieved an improved PCE of 6.54% by using CH_3_NH_3_PbI_3_ in DSSCs, in 2011 [2]. In 2012, CH_3_NH_3_PbI_3_ and CH_3_NH_3_PbI_2_Cl were used as absorbers in solid-state hybrid solar cells to obtain PCEs of 9.7% and 10.9%, respectively [3,4], and research to increase the PCE of perovskite solar cells (PSCs) is ongoing [5,6,7,8,9,10,11,12,13,14,15,16,17,18,19,20,21,22,23,24,25,26,27]. In 2017, Seok and coworkers achieved a PCE of PSCs, as high as 22.1% [28].

The quality of the perovskite layer is important for increasing the PCE of PSCs [28,29,30,31,32]. Research to improve the quality of perovskite films by using additives is currently being pursued. For example, the addition of CH_3_NH_3_Cl [33], CaCl_2_ [34], InCl_3_ [35], NH_4_Cl [36,37] etc., has been found to improve the morphology of perovskite films, thereby improving the performance of PSCs. PSCs with Cl-containing additives, showed high electrical conductivity and a long carrier diffusion length. Additives including, I^−^ also affect the performance of PSCs. When NaI [38], CuI [38], HI [39], and NH_4_I [40] were used as additives, the PCEs of the resulting PSCs were 15.14%, 15.25%, 17.60%, and 18.31% respectively, which were higher compared to that of the PSCs without additives. Previous studies have shown that additives improve the morphology of perovskite films and increase the PCE of PSCs.

In this study, NH_4_I, NH_4_Br, and NH_4_Cl were used as additives in the perovskite solution for film formation via spin-coating. The CH_3_NH_3_PbI_3-x_Cl_x_ perovskite solution for spin-coating was prepared in dimethylformamide (DMF). The concentration of the additives was varied, between 0.1 M and 0.3 M. After spin-coating, the perovskite films were annealed at 100 °C for 1, 5, 10, and 30 min. PSCs with an indium tin oxide (ITO)/poly(3,4-ethylenedioxythiophene):polystyrene sulfonate (PEDOT:PSS)/CH_3_NH_3_PbI_3-x_Cl_x_ perovskite/[6,6]-phenyl-C_60_ butyric acid methyl ester (PCBM)/bathocuproine (BCP)/LiF/Al device structure were fabricated. This study mainly shows device optimization through the change of various additives (NH_4_I, NH_4_Br, NH_4_Cl), amounts of additives (0.1 M and 0.3 M), and annealing time (1, 5, 10, and 30 min), respectively. The characteristics of the CH_3_NH_3_PbI_3-x_Cl_x_ PSCs, were evaluated by controlling the annealing time and the concentration of additives, demonstrating that the PCE of the PSCs was improved by the additives. The details of the characteristics of the PSCs and the effects of the additives are discussed herein.

## 2. Experimental Details

### 2.1. Fabrication of CH_3_NH_3_PbI_3-x_Cl_x_ PSCs

**Figure 1:**
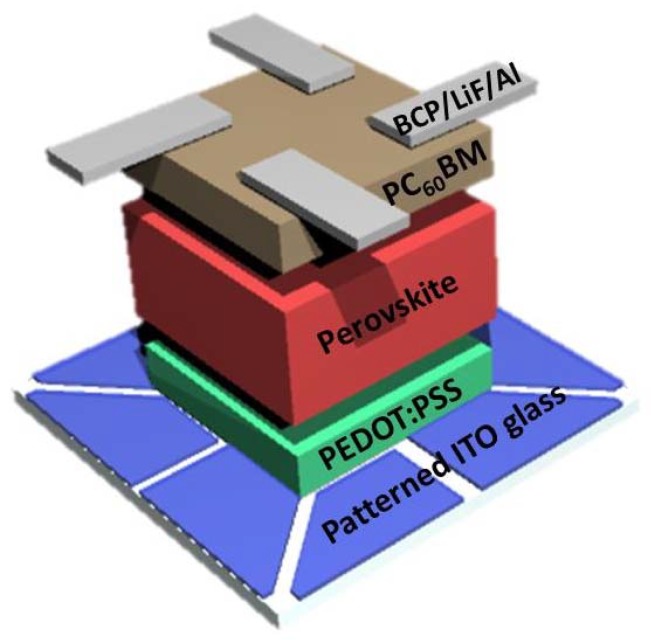
Schematic of inverted planar perovskite solar cells (PSC) device. The PSC is composed of ITO/PEDOT:PSS/CH_3_NH_3_PbI_3-x_Cl_x_/PC_60_BM/BCP/LiF/Al. The single ITO substrate comprised four pixels and the illuminated areas of each pixel were 0.04 cm^2^.

ITO-coated glass (Woo Yang GMS, 15 Ω sq^−1^) was used as the substrate. The glass was cleaned by successive ultrasonication in acetone, isopropyl alcohol, and deionized water for 15 min each. The substrate was then treated with UV-ozone for 15 min. A hole transport layer of PEDOT:PSS (Clevious) was spin-coated onto the glass substrate at 4000 rpm for 30 s, followed by heating at 150 °C for 15 min in air. The substrates were loaded into a N_2_-filled glove box. As for synthesis of the CH_3_NH_3_PbI_3-x_Cl_x_ precursor, CH_3_NH_3_I, lead(II) iodide (PbI_2_), and lead(II) chloride (PbCl_2_) in a 4:1:1 molar ratio, were dissolved in an anhydrous DMF. Thereafter, 0.1 M or 0.3 M NH_4_I, NH_4_Br, and NH_4_Cl were added to the solution. The precursor solution was then spin-coated onto the PEDOT:PSS layer at 4000 rpm for 30 s. The substrates were subsequently annealed at 100 °C for 1, 5, 10, and 30 min. A PCBM (Nanoholdings) solution (40 mg mL^−1^) in chlorobenzene was then spin-coated on top of the perovskite film at 750 rpm for 15 s and the substrate was heated at 60 °C for 5 min. Subsequently, films of BCP (3 nm, Taewon Scientific CO, Seoul, Korea), LiF (1 nm, Taewon Scientific CO, Seoul, Korea), and Al (100 nm) were deposited on top of the electron transport layer (PCBM layer) under vacuum (<10^−6^ Torr) using a thermal evaporator, as shown in Figure 1. BCP and LiF, were used as hole blocking layers and Al was used as an electrode.

### 2.2. Characterizations

The X-ray diffraction (XRD, D8-Advance/Bruker-AXS (Billerica, MA, USA)) patterns were measured in an angle range of 2*θ* = 10° to 50°. The morphology of the films was shown through field emission scanning electron microscope (FE-SEM, Carl Zeiss, SIGMA (Kawasaki-shi, Japan)) images. The current-voltage (*J-V*) characteristics of PSCs were measured with a Keithley 2400 semiconductor parameter analyzer. The measurements were conducted under AM 1.5 G 100 mW/cm^2^ illumination using an Oriel 150 W solar simulator. All devices were measured by masking the active area with a thin mask (0.04 cm^2^). The *J-V* characteristics for all devices were measured at a voltage scan rate of 0.1 V s^−1^.

## 3. Results and Discussion

**Figure 2:**
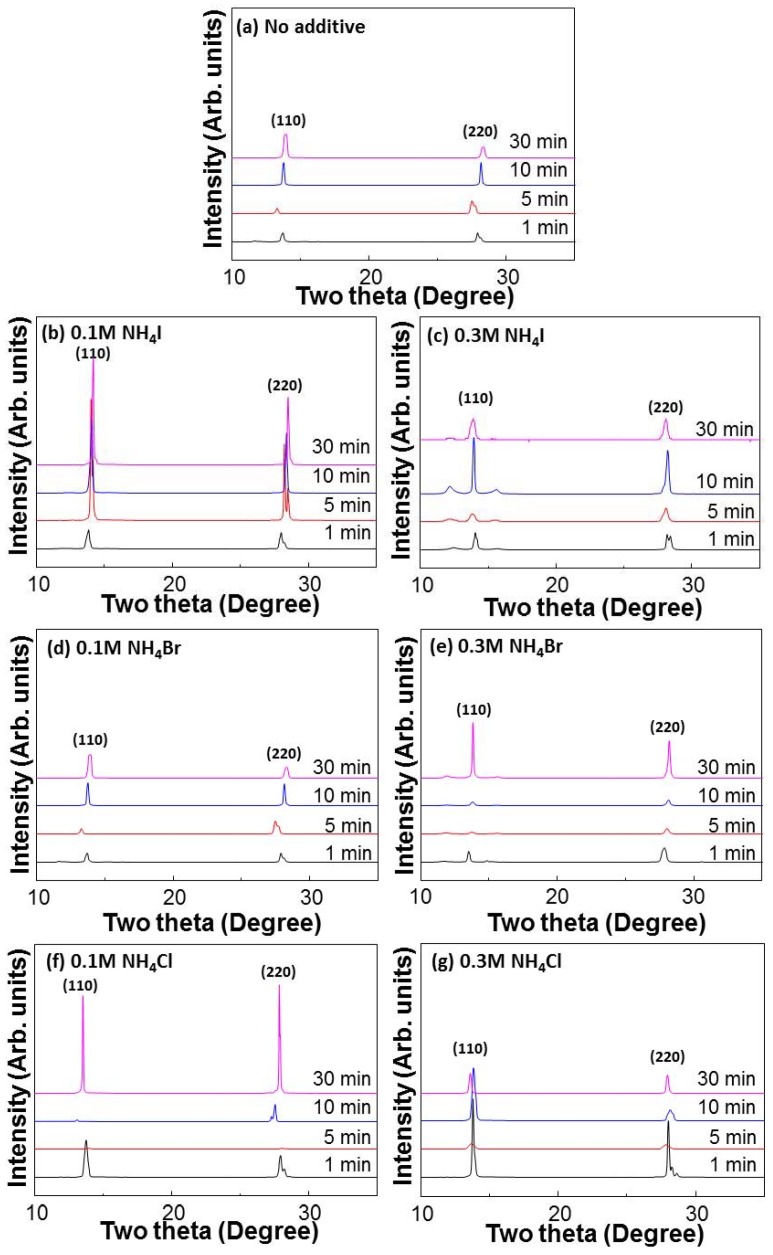
XRD spectra of CH_3_NH_3_PbI_3-x_Cl_x_ layers spin-coated on PEDOT:PSS layers. The CH_3_NH_3_PbI_3-x_Cl_x_ solutions used for spin-coating contained (**a**) no additive, (**b**) 0.1 M NH_4_I, (**c**) 0.3 M NH_4_I, (**d**) 0.1 M NH_4_Br (**e**) 0.3 M NH_4_Br (**f**) 0.1 M NH_4_Cl, and (**g**) 0.3 M NH_4_Cl respectively. The samples were annealed at 100 °C for different times (1, 5, 10, 30 min).

We investigated the effect of the additives on crystallization of the CH_3_NH_3_PbI_3-x_Cl_x_ film through XRD, as shown in Figure 2. The perovskite films were prepared on PEDOT:PSS-coated ITO glass. The samples with and without additives were annealed at 100 °C for various times. NH_4_I, NH_4_Br, and NH_4_Cl were used as additives. The XRD peaks of the CH_3_NH_3_PbI_3-x_Cl_x_ film are known to occur at 14° and 28° [36], as confirmed in Figure 2a. The XRD profiles of CH_3_NH_3_PbI_3-x_Cl_x_ without additives annealed for 1 min and 30 min, showed peaks at 13.8° and 28.0°. The samples annealed for 5 min and 10 min, showed peaks at 14.0° and 28.3°, and 14.1° and 28.4°, respectively. The sample annealed for 5 min exhibited the most intense peaks, indicating that the annealing time affected crystallization of the perovskite. The sample prepared with 0.1 M NH_4_I and annealed for 5 min, also showed strong peaks at 14° and 28° (Figure 2b). Figure 2c shows the XRD peaks of the perovskite film prepared using 0.3 M NH_4_I. Weak peaks were observed with annealing for 5 min and 30 min, but strong peaks were observed for the sample annealed at 10 min. However, the XRD peak of PbI_2_ was observed at 12° [41] for the samples annealed for 1 min and 10 min. The peak of PbI_2_ treated with 0.1 M NH_4_Br and 0.3 M NH_4_Br, is also shown in Figure 2d,e. The layers treated with NH_4_Cl also showed XRD peaks at 14° and 28° (Figure 2f,g). The sample treated with 0.1 M NH_4_Cl and annealed for 30 min exhibited a strong peak, whereas the samples annealed for 5 and 10 min exhibited a weak peak at 14°. All samples showed strong or weak peaks at 14° and 28°. Compared to, without additive, the XRD peaks of layers with additive showed strong peaks in all the layers, except for using 0.3 M NH_4_I and 0.3 M NH_4_Cl when annealed for 30 min. The layers using 0.1 M NH_4_I, 0.3 M NH_4_I, and 0.3 M NH_4_Cl showed a stronger peak than the layer without additive, whilst the layers using 0.1 M NH_4_Br, 0.3 M NH_4_Br, and 0.1 M NH_4_Cl showed weak peaks. All layers except 0.1 M NH_4_I showed weaker peaks than the layer without additive, when annealed for 5 min. In the case of annealing for 1 min, all layers except 0.1 M and 0.3 M NH_4_Cl showed a peak similar to the layer without additive. The samples treated with NH_4_Cl showed a stronger peak than the sample without additive. Thus, it was found that the annealing time and the additives influenced crystallization of the CH_3_NH_3_PbI_3-x_Cl_x_ films.

**Figure 3:**
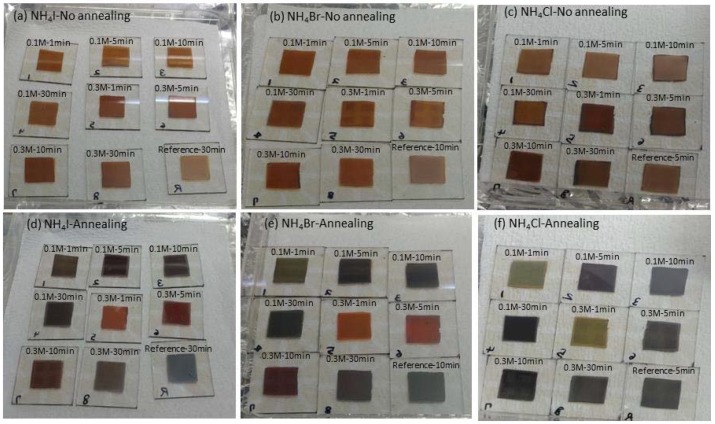
Optical images of CH_3_NH_3_PbI_3-x_Cl_x_ layers prepared with (**a**) NH_4_I, (**b**) NH_4_Br, and (**c**) NH_4_Cl before annealing process and (**d**) NH_4_I, (**e**) NH_4_Br, (**f**) NH_4_Cl after annealing process. The color change occurred after annealing.

Color changes in the perovskite layers were observed according to the annealing time and the additives. The CH_3_NH_3_PbI_3-x_Cl_x_ solution was combined with different concentrations of NH_4_I, NH_4_Br, or NH_4_Cl as additives for spin coating. The samples were annealed at 100 °C for 1, 5, 10, or 30 min. Figure 3 shows optical images of the CH_3_NH_3_PbI_3-x_Cl_x_ layers, before and after annealing. Before annealing, the CH_3_NH_3_PbI_3-x_Cl_x_ films were brown, as shown in Figure 3a–c. There was only a slight difference depending on the additives. The color of the CH_3_NH_3_PbI_3-x_Cl_x_ films changed after annealing. As shown in Figure 3d, the samples prepared with 0.1 M NH_4_I were nearly black regardless of the annealing time. However, those prepared with 0.3 M NH_4_I were red and were similar to the layers prepared with 0.3 M NH_4_Br (Figure 3e). After annealing the perovskite layer with 0.1 M NH_4_Br for 1 min, the layer became green. However, the layer with 0.1 M NH_4_Cl was a lighter green than that prepared with 0.1 M NH_4_Br and annealed for 1 min. With the use of the 0.1 M additives, the color was similar to that of the reference cells after heat treatment. Therefore, it was confirmed that a small amount of additive had no significant effect on color. However, the effect of the annealing time in the crystallization of the perovskite could be confirmed through the different color of perovskite layers, depending on the annealing time. The color of the perovskite layers treated with 0.3 M additives appeared to be affected by I^−^, Br^−^, and Cl^−^, respectively. The use of 0.3 M NH_4_Br and NH_4_I with 1 min of annealing produced similar color changes, whereas NH_4_Cl induced the formation of a greenish color. When the layers treated with 0.3 M additives were annealed for 5 min, those treated with NH_4_I and NH_4_Br were red, whereas the use of NH_4_Cl produced a black phase, similar in color to the perovskite layer without additives. The use of 0.3 M additives and a longer annealing time gave rise to a black phase, similar to the CH_3_NH_3_PbI_3-x_Cl_x_ layer without additives.

**Figure 4:**
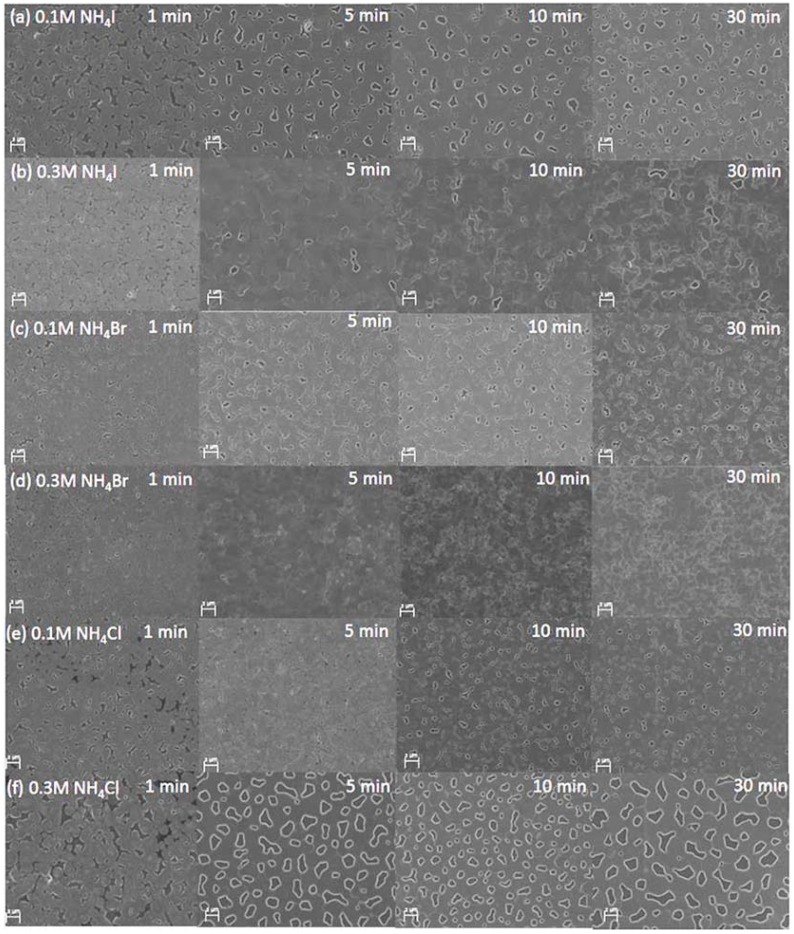
FE-SEM images of CH_3_NH_3_PbI_3-x_Cl_x_ layers coated on PEDOT:PSS layers. The CH_3_NH_3_PbI_3-x_Cl_x_ solutions used for spin-coating contained (**a**) 0.1 M NH_4_I, (**b**) 0.3 M NH_4_I, (**c**) 0.1 M NH_4_Br, (**d**) 0.3 M NH_4_Br, (**e**) 0.1 M NH_4_Cl, and (**f**) 0.3 M NH_4_Cl, respectively. The samples were annealed at 100 °C for different times (1, 5, 10, and 30 min). The scale bar is 2 µm.

The color of the CH_3_NH_3_PbI_3-x_Cl_x_ layers, is illustrated in Figure 3. Notably, the surface quality and morphology have more influence on the performance of CH_3_NH_3_PbI_3-x_Cl_x_ PSCs, than the color of the perovskite layer [42]. The CH_3_NH_3_PbI_3-x_Cl_x_ films with additives were prepared on a PEDOT:PSS film. Figure 4 shows FE-SEM images of the perovskite layers annealed for different times, with different additives. When 0.1 M NH_4_I was added to the CH_3_NH_3_PbI_3-x_Cl_x_ solution, the grain size increased and there were some pinholes compared to that of the sample annealed for 1 min (Figure 4a). Figure 4b shows that when 0.3 M NH_4_I was added, the grain size increased as the annealing time increased, and annealing for 30 min resulted in grains that were clustered with each other. As shown in Figure 4c,d, the FE-SEM images of the perovskite layer prepared with NH_4_Br as the additive, showed an uneven surface. Particularly, the image of the sample prepared with 0.3 M NH_4_Br showed a bright spot, which is estimated to be PbI_2_, as confirmed by XRD. When NH_4_Cl was used as the additive, there were many differences in the FE-SEM images depending on the concentration. Comparison of the samples prepared with 0.1 M NH_4_Cl (Figure 4e) and 0.3 M NH_4_Cl (Figure 4f), shows that there were large pinholes in the film prepared with 0.3 M NH_4_Cl, which is expected to have a negative effect on the perovskite layer as the light absorbing layer in solar cells.

**Figure 5:**
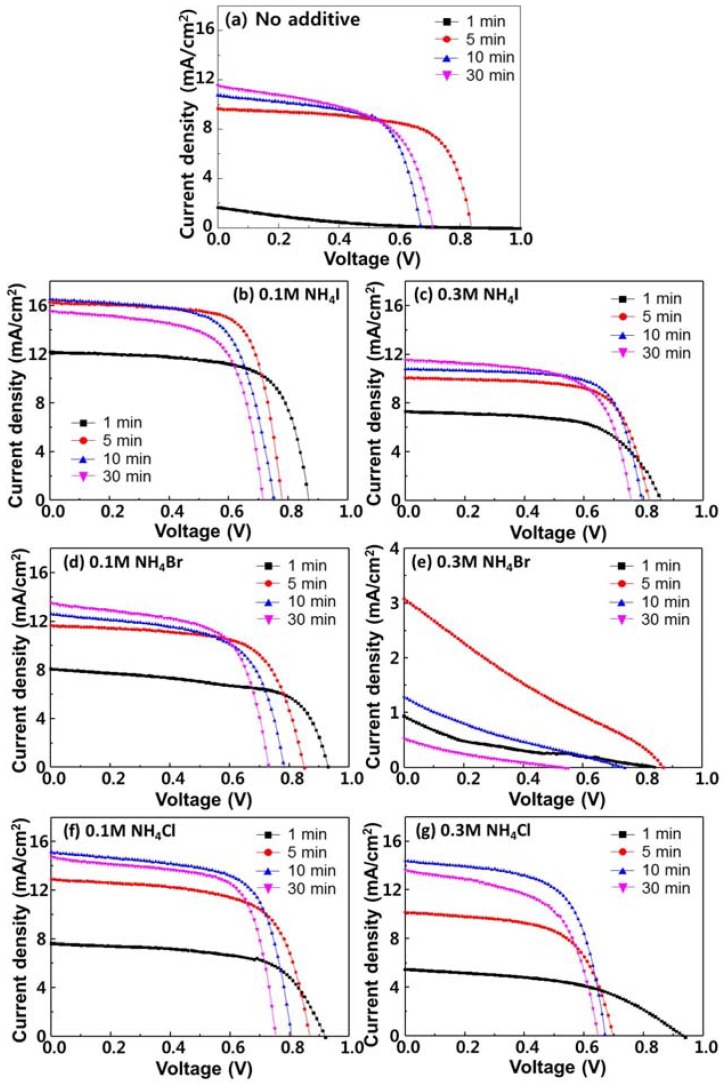
(**a**) Current density-voltage curves of CH_3_NH_3_PbI_3-x_Cl_x_ PSCs, without any additives. The samples were annealed at 100 °C for 1, 5, 10, and 30 min, respectively. The optimal annealing time was 10 min with PCE 5.40%. Current density-voltage curves of CH_3_NH_3_PbI_3-x_Cl_x_ PSCs with additives of (**b**) 0.1 M NH_4_I, (**c**) 0.3 M NH_4_I, (**d**) 0.1 M NH_4_Br, (**e**) 0.3 M NH_4_Br, (**f**) 0.1 M NH_4_Cl, and (**g**) 0.3 M NH_4_Cl, respectively. The maximum average PCE values of the CH_3_NH_3_PbI_3-x_Cl_x_ PSCs with NH_4_I, NH_4_Br, and NH_4_Cl additives are 9.13, 6.57, and 8.13%, respectively.

Figure 5 shows the current density-voltage (*J-V*) curves of the CH_3_NH_3_PbI_3-x_Cl_x_ PSCs with NH_4_I, NH_4_Br, and NH_4_Cl as additives. As a reference, a CH_3_NH_3_PbI_3-x_Cl_x_ PSC without any additive was also fabricated, as shown in Figure 5a. The open-circuit voltage (*V_OC_*), short-circuit current (*J_SC_*), fill factor (FF), and power conversion efficiency (PCE) of the PSCs employing the films with additives, are summarized in Table 1. The perovskite layers were annealed at 100 °C for different times. The annealing times were 1, 5, 10, and 30 min. For the reference CH_3_NH_3_PbI_3-x_Cl_x_ PSCs, the highest PCE of 5.40%, was achieved with the film annealed for 5 min. The corresponding *J_SC_*, *V_OC_*, and FF were 0.84, 9.45, and 0.68, respectively. The same PCE (4.64%) was achieved with annealing times of 10 min and 30 min. On the other hand, when the CH_3_NH_3_PbI_3-x_Cl_x_ layer was annealed for 1 min, the *J_SC_*, *V_OC_*, FF, and PCE values declined significantly to 1.64 mA cm^−2^, 0.81 V, 0.15, and 0.20%, respectively. Thus, it could be deduced that the cubic perovskite was not formed when the perovskite layer was annealed for 1 min. However, the PCE of the CH_3_NH_3_PbI_3-x_Cl_x_ PSCs employing the films with additives was higher than that of the PSCs employing the films without additives annealed for 1 min. The *V_OC_*, *J_SC_*, FF, and PCE of 0.86 V, 12.2 mA cm^−2^, 0.67, and 7.31%, respectively achieved using 0.1 M NH_4_I were significantly higher values. When 0.1 M NH_4_I was used as the additive and the perovskite layer was annealed for 5 min, the highest efficiency was obtained. The corresponding *V_OC_*, *J_SC_*, FF, and PCE values were 0.78 V, 16.3 mA cm^−2^, 0.72, and 9.13%, respectively. The PCE was 69% higher than that of the PSCs employing the perovskites without additives, under the same conditions. It was confirmed that the PCE decreased with longer heat treatment. When the concentration of NH_4_I was increased to 0.3 M, the PSC performance declined. Unlike the case with 0.1 M NH_4_I, where the highest PCE was achieved with the film annealed for 5 min, the highest PCE attained with 0.3 M NH_4_I was achieved with annealing for 10 min. The corresponding *V_OC_*, *J_SC_*, FF, and PCE were 0.79 V, 10.8 mA cm^−2^, 6.70, and 6.06%, respectively. Thus, it was confirmed that the higher the concentration of NH_4_I as the additive, the longer the required annealing time. The CH_3_NH_3_PbI_3-x_Cl_x_ PSCs with the NH_4_Br-treated film showed remarkably low performance when 0.3 M NH_4_Br was added, compared to 0.1 M NH_4_Br. The *V_OC_*, *J_SC_*, FF, and PCE values of the PSCs employing the 0.1 M NH_4_Br-treated film were 0.85 V, 11.7 mA cm^−2^, 0.66, and 6.57%, respectively, whereas that of the PSCs with the 0.3 M NH_4_Br-treated films were 0.87 V, 3.09 mA cm^−2^, 0.23, and 0.61%, respectively, when annealing was performed for 5 min. When 0.1 M NH_4_Br was used as the additive, the Br^−^ in NH_4_Br did not affect the CH_3_NH_3_PbI_3-x_Cl_x_ structure significantly. However, when 0.3 M NH_4_Br was added, it is considered that Br^−^ bonded with CH_3_NH_3_PbI_3-x_Cl_x_ and disrupted the cubic structure. On the other hand, the performance parameters of the PSCs were higher when NH_4_I and NH_4_Cl were used as additives. It is considered that I^−^ and Cl^−^ helped to maintain the cubic structure of the perovskite. The *V_OC_*, *J_SC_*, and FF were 0.80 V, 15.1 mA cm^−2^, and 0.67, respectively, for the highest PCE of 8.13%, achieved with 0.1 M NH_4_Cl. With the use of 0.3 M NH_4_Cl, the *V_OC_*, *J_SC_*, FF, and PCE values were 0.67 V, 14.4 mA cm^−2^, 0.63, and 6.13%, respectively. The performance of the CH_3_NH_3_PbI_3-x_Cl_x_ PSCs employing the films treated with NH_4_I, NH_4_Br, and NH_4_Cl as additives was investigated. The additive is related to the grain size of perovskite. As a result, the diffusion length of the active layer became longer as the grain size increased, confirming that *J_SC_* and PCE were increased. It can also be seen that a small amount of additive was more effective than a large amount. In addition, it was confirmed that the annealing time affected the characteristics of the perovskite solar cells due to the decrease in efficiency, as the annealing time became longer.

## 4. Conclusions

In summary, an innovative approach by adding the methylammonium halide to enhance the performance of CH_3_NH_3_PbI_3-x_Cl_x_ PSCs was developed. CH_3_NH_3_PbI_3-x_Cl_x_ films were fabricated for one-step coating with perovskite solution, using NH_4_I, NH_4_Br, and NH_4_Cl as additives. The XRD patterns and FE-SEM images of the CH_3_NH_3_PbI_3-x_Cl_x_ films with additives generally, showed the presence of strong peaks at 14° and 28°, and an increase in the grain size of the perovskite with additives, but some pinholes were present. Investigation of the photovoltaic performance of the PSCs with additives, showed that the PCE of the CH_3_NH_3_PbI_3-x_Cl_x_ PSCs with the 0.3 M NH_4_Br-treated films was lower than that of the PSCs employing films without additives. It is proposed that, Br^−^ from NH_4_Br substituted the I^−^ of CH_3_NH_3_PbI_3-x_Cl_x_, thereby disrupting its structure, and I^−^ reacted with Pb^+^ to form PbI_2_, leading to a decrease in the PCE. The highest PCE of the PSCs employing the films treated with 0.1 M NH_4_I was 9.13%, which was 69% higher than that of the PSCs employing films without any additive. It was confirmed that small amounts of NH_4_I, NH_4_Br, and NH_4_Cl maintain structure and improve the properties of CH_3_NH_3_PbI_3-x_Cl_x_ PSCs. These results indicated that the additives, especially 0.1 M NH_4_I, were effective in improving the characteristics of CH_3_NH_3_PbI_3-x_Cl_x_ PSCs.

## Figures and Tables

**Table 1:** Photovoltaic performance of CH_3_NH_3_PbI_3-x_Cl_x_ PSCs with films treated with NH_4_I, NH_4_Br, and NH_4_Cl additives. The average values are from 20 cells for each type of device, with AM 1.5 G solar irradiation.

Additives	Annealing Time (min)	*V_OC_* (V)	*J*_SC_ (mA/cm^2^)	FF	PCE (%)
**NH_4_I, 0.1 M**	1	0.86	12.2	0.67	7.31
	5	0.78	16.3	0.72	9.13
	10	0.75	16.5	0.66	8.20
	30	0.71	15.6	0.66	7.31
**NH_4_I, 0.3 M**	1	0.86	7.31	0.62	3.87
	5	0.82	10.1	0.69	5.72
	10	0.79	10.8	0.70	6.06
	30	0.76	11.6	0.65	5.67
**NH_4_Br, 0.1 M**	1	0.93	8.08	0.62	4.69
	5	0.85	11.7	0.66	6.57
	10	0.78	12.6	0.62	4.69
	30	0.73	13.6	0.63	6.23
**NH_4_Br, 0.3 M**	1	0.91	0.91	0.19	0.15
	5	0.87	3.09	0.23	0.61
	10	0.73	1.29	0.20	0.19
	30	0.53	0.53	0.18	0.051
**NH_4_Cl, 0.1 M**	1	0.92	7.63	0.63	4.42
	5	0.87	12.9	0.65	7.30
	10	0.80	15.1	0.67	8.13
	30	0.75	14.8	0.68	7.56
**NH_4_Cl, 0.3 M**	1	0.93	5.25	0.51	2.49
	5	0.70	10.2	0.61	4.36
	10	0.67	14.4	0.63	6.13
	30	0.64	13.1	0.60	5.11
**No additive**	1	0.81	1.64	0.15	0.20
	5	0.84	9.45	0.68	5.40
	10	0.67	10.8	0.64	4.64
	30	0.71	11.6	0.56	4.64
